# Promoter hypermethylation of neural-related genes is compatible with stemness in solid cancers

**DOI:** 10.1186/s13072-023-00505-7

**Published:** 2023-08-03

**Authors:** Musa Idris, Louis Coussement, Maria M. Alves, Tim De Meyer, Veerle Melotte

**Affiliations:** 1 Department of Pathology, GROW-School for Oncology and Developmental Biology, Maastricht University Medical Centre, P.O. Box 616, 6229 HX Maastricht, The Netherlands; 2 Department of Clinical Genetics, Erasmus University Medical Center-Sophia Children’s Hospital, 3015 GD Rotterdam, The Netherlands; 3 Department of Data Analysis and Mathematical Modelling, Ghent University, 9000 Ghent, Belgium; 4 Cancer Research Institute Ghent (CRIG), Ghent University, 9000 Ghent, Belgium

**Keywords:** DNA hypermethylation, Pan cancer, Neural differentiation, REST

## Abstract

**Background:**

DNA hypermethylation is an epigenetic feature that modulates gene expression, and its deregulation is observed in cancer. Previously, we identified a neural-related DNA hypermethylation fingerprint in colon cancer, where most of the top hypermethylated and downregulated genes have known functions in the nervous system. To evaluate the presence of this signature and its relevance to carcinogenesis in general, we considered 16 solid cancer types available in The Cancer Genome Atlas (TCGA).

**Results:**

All tested cancers showed significant enrichment for neural-related genes amongst hypermethylated genes. This signature was already present in two premalignant tissue types and could not be explained by potential confounders such as bivalency status or tumor purity. Further characterization of the neural-related DNA hypermethylation signature in colon cancer showed particular enrichment for genes that are overexpressed during neural differentiation. Lastly, an analysis of upstream regulators identified RE1-Silencing Transcription factor (REST) as a potential mediator of this DNA methylation signature.

**Conclusion:**

Our study confirms the presence of a neural-related DNA hypermethylation fingerprint in various cancers, of genes linked to neural differentiation, and points to REST as a possible regulator of this mechanism. We propose that this fingerprint indicates an involvement of DNA hypermethylation in the preservation of neural stemness in cancer cells.

**Supplementary Information:**

The online version contains supplementary material available at 10.1186/s13072-023-00505-7.

## Background

Cancer research has advanced rapidly, but the causal mechanisms and pathways of carcinogenesis are still incompletely understood. Cancer originates from genetic and epigenetic alterations that confer adaptive advantages to cells in terms of fitness, survival, and proliferation [1]. These (epigenetic) modifications lead to aberrant activation or silencing of specific signaling pathways out of temporal and spatial contexts, granting potency and bringing potential alterations in cellular identity [2]. Moreover, cancer cells interact with and exploit their microenvironment, including immune cells, fibroblasts, and neural cells. The role of the nervous system in solid cancer development has only recently gained increasing attention [3–5]. Bidirectional communication between neural cells and cancer cells has been described, which impacts tumor development, growth, and therapeutic resistance. Cancer cells form synapses with neurons and release neurotransmitters, neurotrophins, or vesicles that enhance their survival and metastatic ability [4, 6, 7]. Cancer cells can also stimulate neuronal growth and recruit distant neural stem cells to invade and innervate the tumor mass [3]. Also, cancer cells use nerve fibers as rails and guides for migration and dissemination [4].

Interestingly, the concept of “neural signature in cancer” extends beyond such crosstalk. Cancer cells promote their fitness by activating intracellular programs that are typical for neural stem cells [8]. For example, cancer cells may differentiate into neuron-like cells in vitro and in vivo. Although these differentiated cells are post mitotic and less able to form tumors, they can promote the progression of colon and gastric cancer [8, 9]. Similarly, cancer cells utilize neural crest cell abilities to move and invade [10], as neural stemness is essential for their tumorigenic ability [9, 11]. Conversely, inducing cancer cells to differentiate away from the neural lineage can suppress their tumorigenicity [12]. However, the underlying mechanism regulating this process remains elusive. Previous studies have reported that 29% of established oncogenes are embryonic neural-specific genes, while tumor suppressor genes are less enriched within these genes [13]. Recently, we have shown that a significant fraction of hypermethylated genes in colon adenocarcinoma are neural-related [14]. Based on these findings, we hypothesized that hypermethylation of neural-related genes can maintain a neural stemness state in cancer cells and promote carcinogenesis.

In this manuscript, we verified that DNA hypermethylation of neural-related genes not only occurs in colon cancer, but is a pan cancer signature, i.e., is present in all solid cancers included in The Cancer Genome Atlas (TCGA). Subsequently, we investigated to which extent DNA hypermethylation during carcinogenesis affects genes involved in neural differentiation. Finally, we performed an upstream regulators analysis to identify DNA-binding proteins that are plausibly associated with the DNA-methylation status in these genes. Our work proposes a neuro-methylation signature via which cancer cells hijack a neural stemness-like state during carcinogenesis.

## Results

### Promoter hypermethylation of neural-related genes is a common feature of solid cancers

**Fig. 1:**
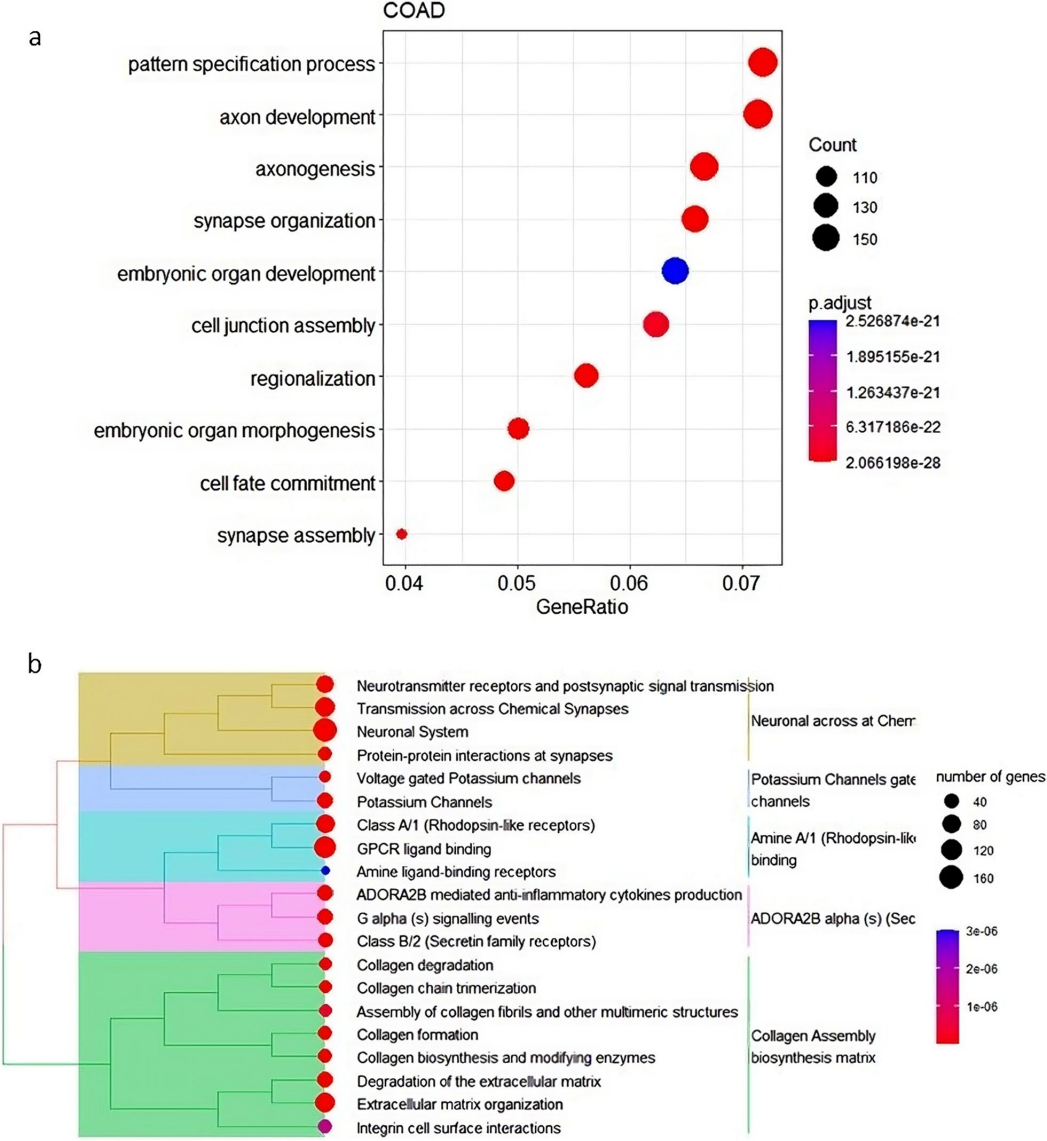
Enrichment of neural terms in hypermethylated genes in cancer. Panel A shows an enrichment map plot of GO enriched terms for hypermethylated genes in COAD. Panel B shows a tree plot of the top Reactome pathways enriched for hypermethylated genes in COAD

To explore neural-related DNA hypermethylation in different cancers, we first performed differential DNA methylation analysis on the 144,004 probes that are located in the promoter region (29.7% of the total Infinium Methylation 450k Bead Array probes), corresponding to 21,089 Ensembl gene ids. Throughout this manuscript, we first explore results in colon adenocarcinoma (COAD), followed by a pan cancer analysis. We identified 11,659 hypermethylated promoter probes in COAD, resulting in 2624 genes with at least one hypermethylated probe in their promoter (logFC > 1, adjusted *P* < 0.05, and mean difference in *B*-value > 0.1). Additionally, all tested cancer types featured hypermethylation in about 1700 to 3400 genes, except for thyroid cancer that showed as little as 207 hypermethylated genes (Table 1). Figure 1 shows the top 10 enriched Gene Ontology (GO) terms and the top affected pathways in the 2624 hypermethylated genes observed in COAD compared to normal colon tissue. The lists of significantly differentially methylated probes in all the tested cancer types are provided in Additional file 1: Significant Limma output per cancer type.xlsx.

**Table 1:** Enrichment of neural genes in hyper- and hypomethylated genes for solid cancers

Cancer type	Abbreviation	Number of hypermethylated genes	Percentage of neural gene	FDR	Number of hypomethylated genes	Percentage of neural gene	FDR
Bladder cancer	BLCA	1739	37.3	2.5E−78	6186	18.6	0.99
Breast cancer	BRCA	3163	34.2	4.1E−91	2390	16.2	0.02
Cholangiocarcinoma	CHOL	2548	31.0	9.3E−50	1004	24.5	5.5E−05
Colon adenocarcinoma	COAD	2624	38.5	4.8E−123	3153	16.8	0.04
Esophageal cancer	ESCA	2321	33.2	2.6E−62	2086	16.2	0.02
Head and neck cancer	HNSC	2328	36.6	7.1E−93	3504	16.7	0.02
Kidney clear cell carcinoma	KIRC	1848	33.4	3.2E−53	2205	18.5	0.99
Kidney papillary cell carcinoma	KIRP	2369	25.4	1.6E−15	1156	20.3	0.24
Liver cancer	LIHC	1808	36.7	9.0 E−76	4955	18.6	0.99
Lung Adenocarcinoma	LUAD	1970	39.7	1.2E−109	1714	15.1	0.01
Lung squamous cell carcinoma	LUSC	2060	32.9	4.0E−54	4443	16.9	0.02
Pancreatic cancer	PAAD	1758	41.7	3.6E−118	1197	19.8	0.45
Prostate cancer	PRAD	3357	31.4	1.7E−65	1865	16.0	0.02
Rectal cancer	READ	1750	38.5	1.4E−88	3970	17.3	0.09
Thyroid cancer	THCA	207	30.4	2.0E−05	734	19.9	0.53
Endometrioid cancer	UCEC	2684	31.1	9.7E−53	4978	18.9	0.70

The number of differentially hyper- and hypomethylated genes and percentage of neural-related genes are shown for all solid cancers. The false discovery rate (FDR) column gives Benjamini–Hochberg-adjusted *P* values for the enrichment of neural genes within hyper- and hypomethylation upon assessment with a Chi-squared test, compared to the expected amount based on the Infinium array annotation

**Fig. 2:**
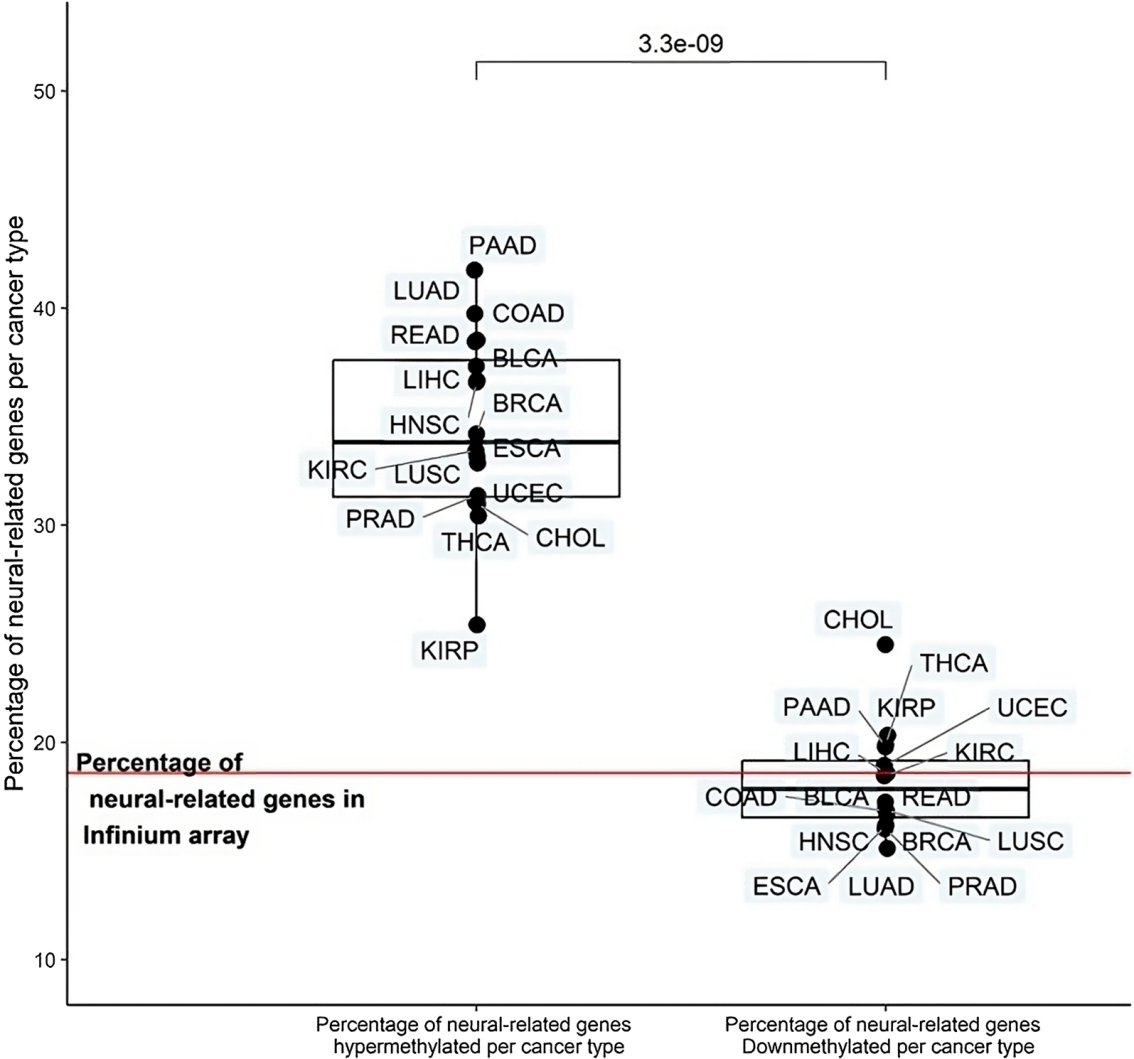
Percentage of neural-related hyper- and hypomethylated genes in different solid cancers. Percentage of neural-related hypermethylated (left) and hypomethylated (right) genes for all analyzed solid cancer types. A paired two-tailed *t*-test was used to compare percentages of neural-related hypomethylated and hypermethylated genes

As described before for COAD [14], hypermethylated genes are enriched with neural-related genes (based on GO, see “Methods”): 38.5% of all promoter hypermethylated genes (*n* = 2624) were flagged as neural-related in COAD, whereas only 18.6% would be expected based on the percentage of neural-related genes on the Infinium array (further referred to as “Background”). Strikingly, this enrichment of neural-related genes amongst hypermethylated genes is consistently present in all cancer types (25.4–41.8%; all FDR-adjusted *P* values < 1.0E−4; Table 1 and Fig. 2), including thyroid cancer. Similar enrichment in all cancer types was found also when only considering DNA methylation in CpG islands into account (Additional file 2: Figures: S1 and S2, Table S1). As for hypomethylation, a similar enrichment of neural-related genes is only seen in cholangiocarcinoma and additionally, 7 out of 16 cancer types even show a derichment of neural-related genes (FDR-adjusted *P* values < 0.05; Table 1 and Fig. 2). We therefore concluded that the relationship with neural-related genes is particularly associated with hypermethylation.

Similarly, Reactome pathway enrichment analysis revealed that the pathway “Neuronal System” (r-has-112316) is enriched in all cancer types. Synaptic and ion channel-related pathways are also commonly enriched (15/16 cancer types). In addition, GO analysis resulted in several neural-related terms to be commonly enriched (15/16 cancer types): cellular component (CC) terms such as axonal components (GO:0043679, GO:0150034) and synaptic membrane components (e.g., GO:0099240, GO:0099572, GO:0097060); biological process (BP) terms such as dopaminergic neuronal differentiation (GO:0071542) and regulation of nervous system development (GO:0051960); and lastly the molecular function (MF) term: postsynaptic neurotransmitter receptor activity (GO: 0098960; Additional file 2: Table S2). Altogether, this analysis showed particular hypermethylation enrichment of neural-related and developmental genes during tumorigenesis. Especially, the neurotransmitters’ dopamine, norepinephrine and serotonin, as well as GABA and glutamate receptor genes were often affected by DNA hypermethylation. A list of all enriched GO terms per cancer type is provided in Additional file 1: GO outcome.rar.

### The neural-related hypermethylation fingerprint is relevant for multiple hallmarks of cancer and is already present in premalignant tissue

**Fig. 3:**
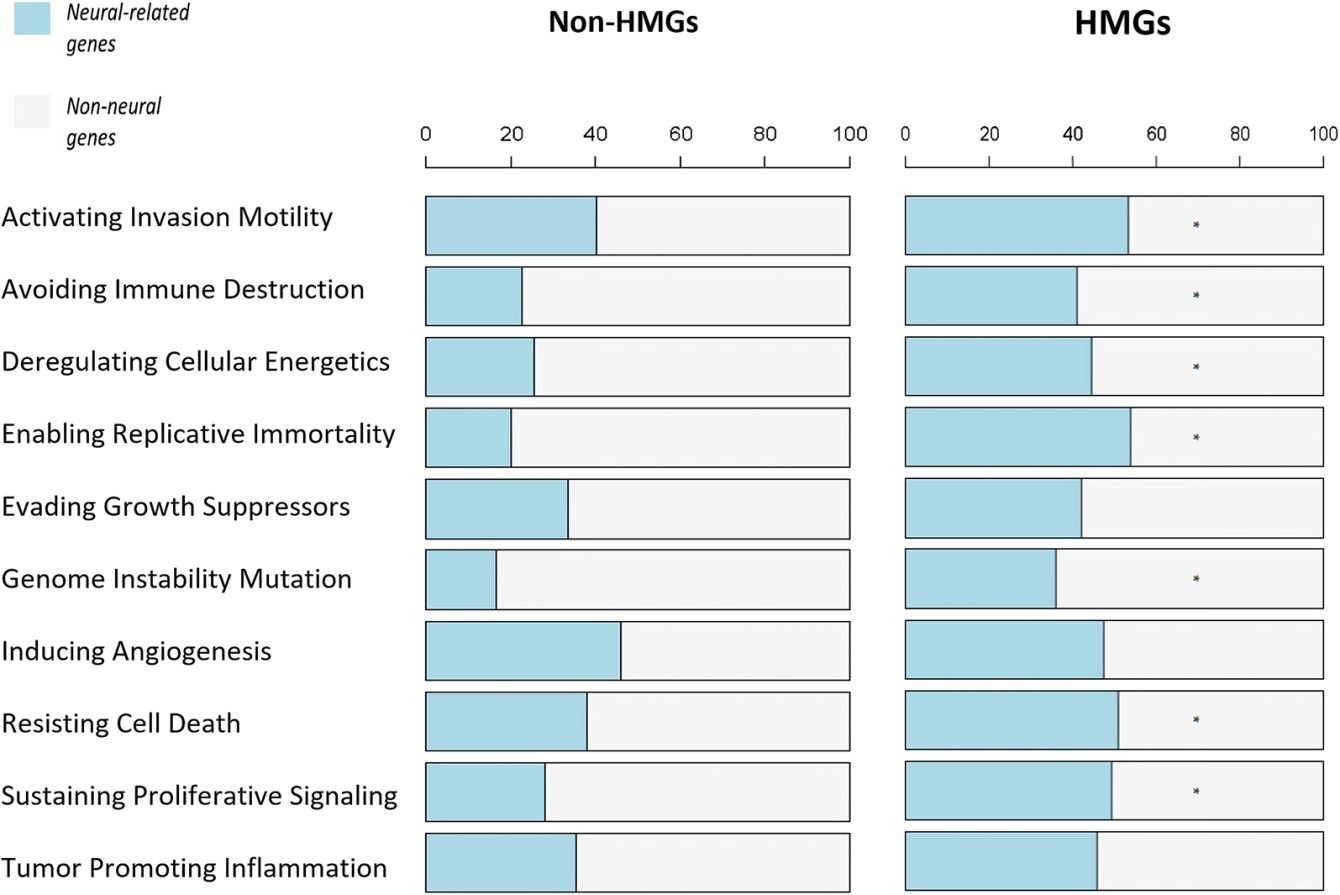
Distribution of neural-related and non-neural-related genes in different hallmarks of cancer. Distribution of neural-related and non-neural-related genes for non-hypermethylated genes (left) and hypermethylated genes (right) for the different hallmarks of cancer

Subsequently, we evaluated to which extent neural-related hypermethylated genes were relevant throughout the hallmarks of cancer. Overlap with gene sets for known hallmarks of cancer [15] showed significant enrichment for neural-related genes amongst differentially hypermethylated genes (HMGs) compared to non-HMGs (FDR < 0.05) for 7 out of 10 studied hallmarks of cancer (Fig. 3, not significant for growth suppressor evasion, angiogenesis induction and tumor promoting inflammation) These results demonstrate that hypermethylation of neural-related genes is relevant for multiple aspects of carcinogenesis.

After establishment of the neural-related fingerprint in a variety of solid cancers, we evaluated whether this signature appears during early carcinogenesis by analyzing premalignant colon and bile duct samples and corresponding controls. 978 (35.8%) HMGs were found to be neural-related in premalignant colon adenoma samples, a significant enrichment compared to the background (Chi-squared test, *P* value < 0.0001). Also, for premalignant bile duct samples a similar enrichment was found, 1316 (32.2%) HMGs were neural-related genes (Chi-squared test, *P*-value < 0.0001). Despite enrichment of neural-related hypermethylated genes in premalignant tissue compared to normal tissue, there is still a significant increase in the enrichment when considering actual cancer tissue—35.8% vs 38.9% in colon cancer (*P* = 0.024) and 32.6% vs 37.0% cholangiocarcinoma (*P* = 0.0003). Of interest, neural genes being hypermethylated in premalignant tissue were typically also hypermethylated in solid cancer (data not shown). These results hint at a progressive mechanism, although a technical origin (e.g., different degrees of tumor purity) cannot be excluded. Therefore, these results indicate that neural-related hypermethylation is a part of the early aberrant DNA methylation events during carcinogenesis that may further progress during tumor development.

### The neural-related fingerprint cannot be explained by tumor purity, bivalency, and higher DNA methylation degrees of neural genes

Tumor purity refers to the percentage of cancer cells in the tumor microenvironment (TME). As the TME consists of several types of cells, including neural cells, enrichment of neural-related genes amongst hypermethylated genes could represent a biological bias, i.e., Due to a different fraction of neural cells in controls and cases. Therefore, we investigated whether the number of hypermethylated genes in a tumor depends on tumor purity for neural-related and non-neural-related genes. For COAD, samples that showed a higher tumor purity displayed a higher fraction of hypermethylated genes (both for neural-related and non-neural-related genes). Therefore, the enrichment of neural-related genes amongst hypermethylated genes is more likely to occur in cancer cells rather than in non-malignant TME cells (Additional file 2: Figure S3), though only microdissection or single cell experiments can provide conclusive proof.

Furthermore, hypermethylated neural-related genes did not feature higher (even slightly lower) methylation degrees in tumor samples than non-neural ones, indicating that the enrichment is not caused by the fact that hypermethylation of neural-related genes is easier to detect (Additional file 2: Figure S4).

Additionally, bivalent genes are known to be more susceptible to hypermethylation in cancer. Therefore, we evaluated the association between the bivalency status in embryonic stem cells (ESCs) and the neural-related status of genes. In conformity with what is known from the literature, we found that hypermethylation of promoter regions occurs preferentially in bivalent genes during carcinogenesis. Indeed, we find bivalent genes to be enriched for hypermethylation in COAD as well as pan cancer (Additional file 2: Figure S5 and Tables S3 and 4). However, hypermethylation of bivalent genes during carcinogenesis was present at very similar (even slightly lower) proportions for neural bivalent genes when compared to non-neural bivalent genes.

### Cancer cells appear to hijack a neural-related stemness state

**Fig. 4:**
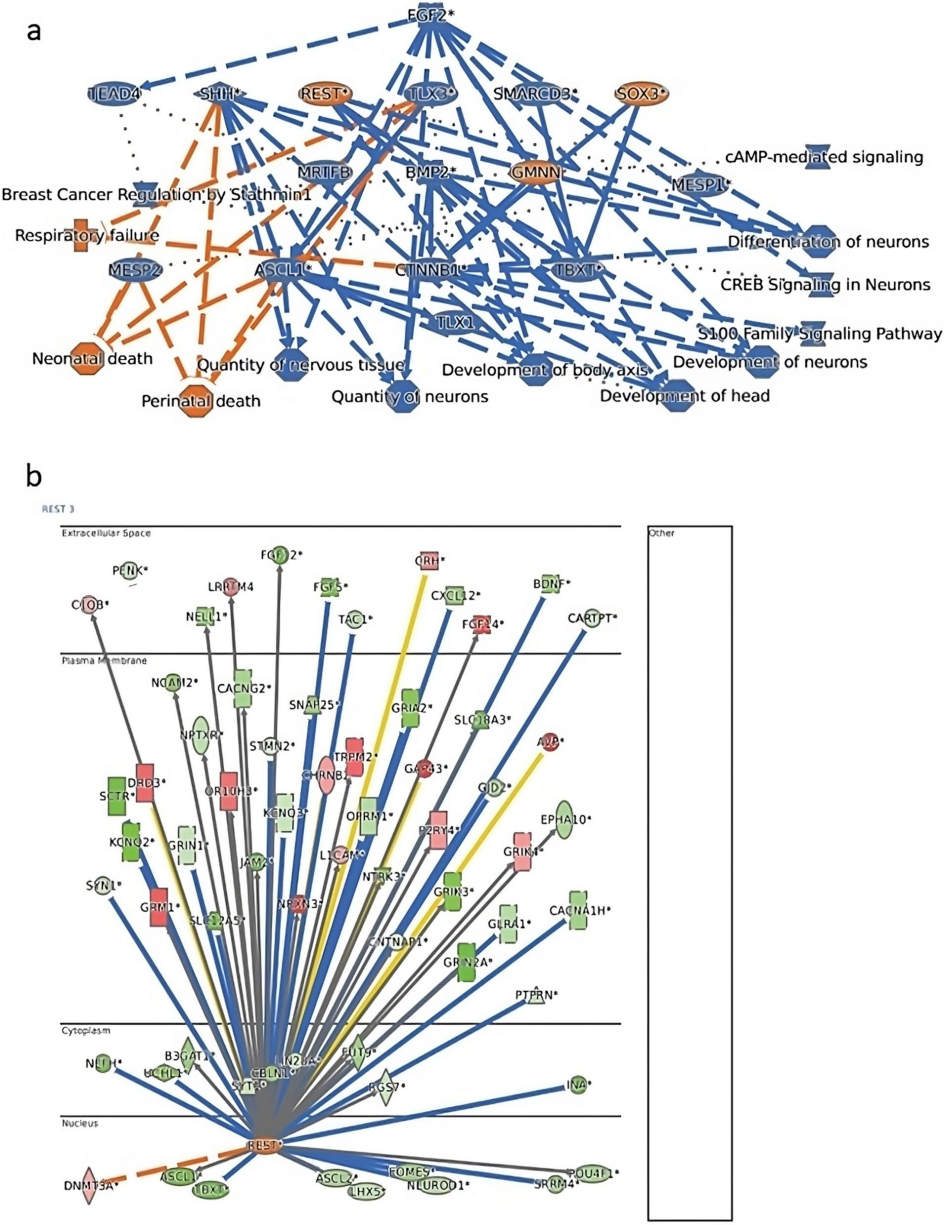
Ingenuity Pathway Analysis (IPA) output summary. **A** An overview network of the major biological themes of the hypermethylated neural-related genes in COAD, and **B** a mechanistic network for the top predicted upstream regulator (REST) and HMGs in COAD

Next, the Ingenuity Pathway Analysis (IPA) software was used to perform an upstream regulators analysis on hypermethylated neural-related genes (vs. non-hypermethylated neural-related genes) to identify possible regulators. Results showed that the master neural specification regulator RE1-Silencing Transcription factor (REST) was the top activated regulator of neural-related promoter hypermethylated genes. This revealed a possible role for REST as mediator of the neural-related hypermethylation fingerprint observed in cancer cells. Figure 4A, B shows mechanistic networks of the top upstream regulator REST revealing predicted pathways that might be involved in this signature such as the WNT pathway. In contrast to REST, differentiation and cell fate specification regulators PTF1A [16], TLX3 [17] and NEUROG2 [18] were marked as inhibited in the IPA analysis, which is consistent with the hypermethylation and subsequent downregulation of neural-related genes (Additional file 2: Table S5). Together with the notion that epigenetics plays a major role in cellular differentiation, the combined enrichment for factors that regulate neural lineage differentiation and specification in this list of upstream regulators hints towards the hypothesis that the neural-related hypermethylation signature helps granting cancer cells the required neural stemness to thrive.

To test our hypothesis, we identified a set of putative neural differentiation-associated genes relying on publicly available expression data, where inducible pluripotent stem cells (iPSCs) were differentiated through neural stem cells into neurons [19]. By performing differential expression analysis on the control samples in their neural stem cell and differentiated neuronal stage, we identified putative neural differentiation-associated genes (*n* = 1083) as those featuring significantly higher expression in differentiated neurons compared to neural progenitor cells. The list of neural differentiation-associated genes is provided in Additional file 1: Neural differentiation-associated genes.xlsx. We found that 29.0% of the differentiation-associated genes were hypermethylated at their promoter regions, vs. 18.6% of all the tested genes in colon cancer (OR = 3.13, *P* value < 0.0001). Moreover, when only considering neural differentiation-associated genes that are also annotated as neural-related by GO terminology, the share of hypermethylated genes increases to 44.1% (177 genes out of 401, OR = 5.88, *P* value < 0.0001).

**Fig. 5:**
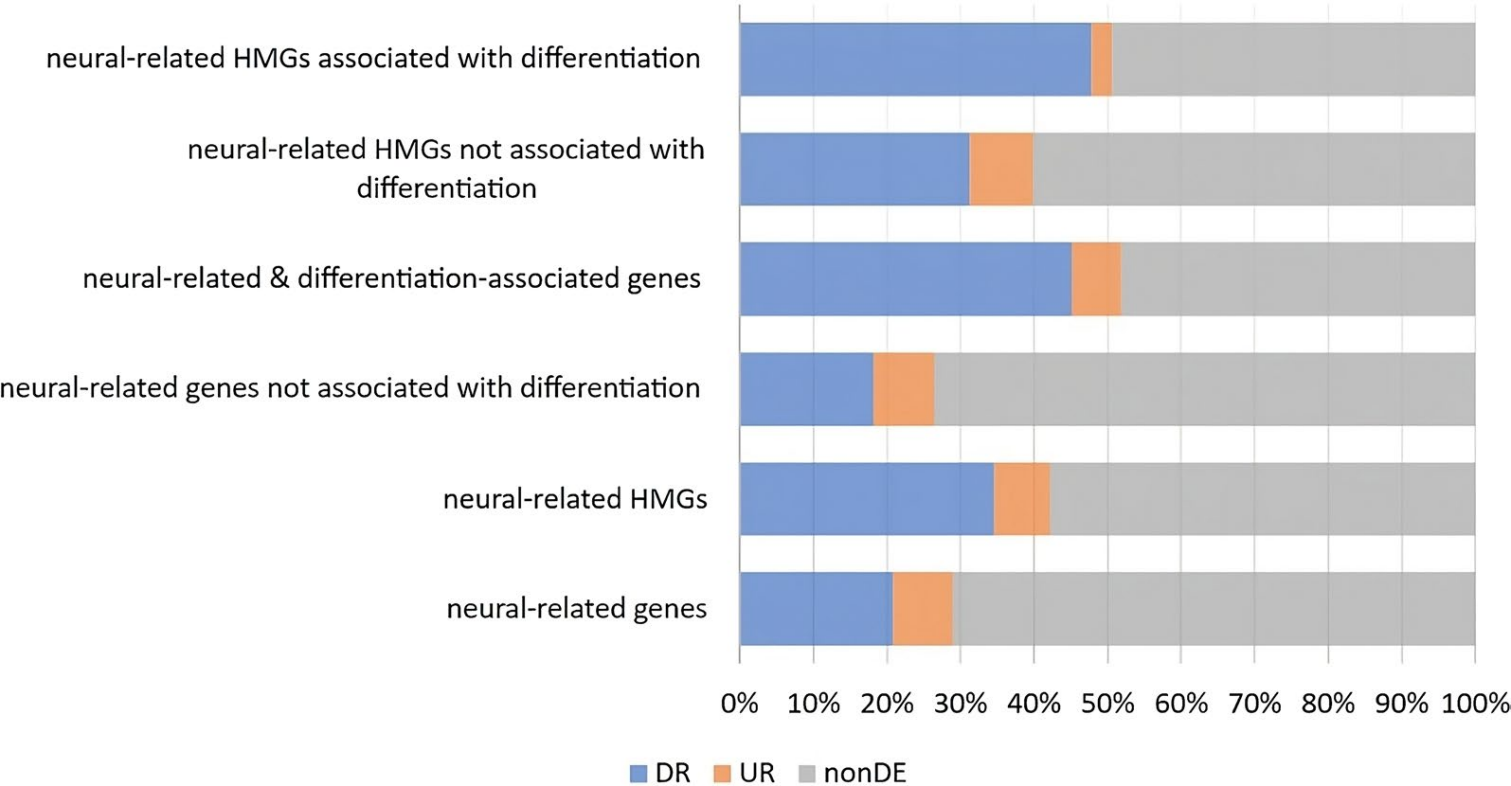
Overview of differential expressed HMG, neural genes, and neural differentiation-associated genes. Fractions of significantly downregulated (DR), upregulated (UR) or non-differentially expressed (nonDE) genes hypermethylated in colon cancer, compared to neural-related and neural differentiation-associated genes

Interestingly, we found that up to 47.8% of these hypermethylated neural and differentiation-related genes, show a significantly reduced expression in colon cancer, versus only 31.3% for hypermethylated neural-related genes that were not overexpressed in differentiated neurons (OR = 2.04, *P* value < 0.0001, Fig. 5).

In summary, these results suggest that hypermethylation of neural differentiation genes might be an active mechanism to silence the expression of neural differentiation genes in colon cancer, thus granting cancer cells neural stemness properties.

## Discussion

We showed that neural-related DNA methylation is a general epigenetic characteristic occurring during (early) carcinogenesis in cancers, confirming earlier studies depicting that neural-related processes are among the most significantly enriched GOs of cancer hypermethylated genes in ovarian [20], colorectal [21–23], bladder [24], adrenal [25], breast [26], cervical [27], bone [28], and lung [29] cancer. Importantly, we demonstrated that these results cannot be readily explained by other biological and technical mechanisms such as tumor purity and bivalency. DNA methylation has been shown to correlate with bivalent modifications on histones during carcinogenesis [30]. A recent study identified that loss of bivalency and concomitant DNA hypermethylation in cancer resembles the same process during the transition to naïve pluripotent stem cells [31]. Here, we showed that neural-related genes are more prone to hypermethylation, both in bivalent and non-bivalent genes in various cancers. However, bivalent genes showed less striking neural-related signature than their counterparts in non-bivalent genes. This may be explained by enhanced de novo DNA methylation activity in neural-related bivalent genes compared to non-neural ones.

Increasing attention has been given to the interaction between the nervous system and tumors [4]. In parallel, similarities between cancer cells and neural progenitors have been discovered, as cancer cells acquiring neural stem-like properties, and suppressing this neural stemness induces tumor cells differentiation [9]. These newly identified characteristics help cancer cells sustain proliferative activities, in addition to facilitating invasion and metastasis. There is evidence that neural-related genes can stimulate cancer cell stemness and potency [10, 13]. Our in silico analysis suggests an involvement of DNA hypermethylation in the preservation of neural stemness in cancer cells. While preserving neural stemness characteristics, cancer cells need to avoid activating neural differentiation programs that will cause cells to exit the cell cycle.

Given the shared methylation status of many neural-related genes, one may hypothesize the relevance of specific factors that recruit or repel the DNA methylation machinery, such as transcription factors that can bind to DNA and chromatin marks and regulate gene expression in a context-dependent manner. We here propose a model where specific DNA marks are recognized by neural-related regulatory factors that are differentially activated or silenced during carcinogenesis. REST is a key transcription factor that controls expression of many neural-related genes. It has a dual role in cancer: it acts as a tumor suppressor in epithelial cancers [32], and as an oncogene in nervous system cancers [33, 34]. Mechanistically, REST interacts with factors involved in establishing DNA methylation such as MECP2 and DNMT1 [35, 36], as well as demethylating factors such as TET1 [37], depending on the cellular context. It also interacts with EZH2, the active component of the polycomb repressive complex (PRC2), which stabilizes REST independently of its role in PRC2. In this study, we identified REST as a putative upstream regulator for the hypermethylation signature of neural-related genes. Previous studies have shown that inhibiting EZH2 or DNMT1 in colon cancer cell lines induces a neural-like differentiation phenotype [13, 38]. Similarly, Lu et al. have shown a similar neural-like differentiation pattern when treating colon cancer cells with vitamin A [8]. Retinoic acid is the active form of vitamin A, and is known to degrade REST and promote neural differentiation in neural stem cells [39]. This implies that EZH2–REST axis may be involved in maintaining neural stemness in cancer cells. In agreement, neural-related hypermethylated genes featured more targets of differentiation and cell fate specification regulators PTF1A, TLX3 and NEUROG2, further supporting the role of hypermethylation in preventing cellular differentiation. IPA upstream regulator analysis on the limited number of hypermethylated, neural and differentiation-related genes showed similar results as for the neural-related genes, yet not significant, probably due to the limited number of genes being analyzed.

The identification of REST as a potential target for modulation of DNA methylation in cancer highlights the importance of understanding the regulatory network that governs neural-related genes in cancer. Future in vitro and in vivo studies are thus needed to unravel the exact mechanism behind this process and its role in cancer formation.

## Conclusions

In summary, in this paper, we suggest a new layer of information by showing that cancer cells seem to actively direct DNA methylation machinery to neural-related genes, to preserve neural stemness. Furthermore, this neural-related hypermethylation fingerprint shows to be relevant in most cancer hallmarks and shows indication to be regulated by the known master neural specification regulator REST. Finally, this mechanism seems to be already present in premalignant state of tumors as well as pan cancer.

## Methods

### Selection of publicly available data

To determine which genes become differentially methylated in various cancer types, TCGA datasets that included normal samples were analyzed: Bladder Cancer (BLCA), Breast Cancer (BRCA), Cholangiocarcinoma (CHOL) Colon Adenocarcinoma (COAD), Endometrioid Cancer (UCEC), Esophageal Cancer (ESCA), Head and Neck Cancer (HNSC), Kidney Clear Cell Carcinoma (KIRC), Kidney Papillary Cell Carcinoma (KIRP), Liver Cancer (LIHC), Lung Adenocarcinoma (LUAD), Lung Squamous Cell Carcinoma (LUSC), Pancreatic Cancer (PAAD), Prostate Cancer (PRAD), Rectal Cancer (READ) and Thyroid Cancer (THCA). For each of these cancer types, we used Infinium Human Methylation 450k BeadChip data to detect differences in methylation between tumor and control samples. Sample sizes per cancer type are available in Table 2. Clinical patient data were used to retrieve the stage and features of the disease for each sample. Analyses that were performed over all cancer types retained from TCGA will typically be referred to as analyses that were run “pan cancer”.

**Table 2:** Infinium Human Methylation 450K BeadChip arrays available in TCGA

Cancer type	Abbreviation	N normal	N tumor
Bladder cancer	BLCA	21	416
Breast cancer	BRCA	96	794
Cholangiocarcinoma	CHOL	9	36
Colon adenocarcinoma	COAD	38	309
Esophageal cancer	ESCA	16	186
Head and neck cancer	HNCA	50	530
Kidney clear cell carcinoma	KIRC	160	323
Kidney papillary Cell carcinoma	KIRP	45	276
Liver cancer	LIHC	50	380
Lung adenocarcinoma	LUAD	32	471
Lung squamous cell carcinoma	LUSC	42	370
Pancreatic cancer	PAAD	10	185
Prostate cancer	PRAD	50	503
Rectal cancer	READ	7	99
Thyroid cancer	THCA	56	515
Endometrioid cancer	UCEC	46	436

Sample sizes for solid cancer types with DNA methylation data available in TCGA

For premalignant colon adenoma samples, we used the publicly available Infinium Human Methylation 450k BeadChip data available on the Gene Expression Omnibus (GEO), under accession number GSE48684 (64 colon cancer samples, 42 adenoma samples and 41 normal samples). For premalignant bile duct samples, we used the publicly available Infinium Human Methylation 450k BeadChip data under accession number GSE156299 (60 premalignant samples and 51 normal samples).

### Differentially hypermethylated genes

All analyses were performed using the R programming language (version 4.2.0). R functions and packages mentioned in methods were used with default settings unless mentioned otherwise. Reported log fold changes (logFC) were all in base 2. For TCGA Infinium Human Methylation 450k BeadChip data, we included all probes within/near the promoter region [i.e., Probes annotated by the terms “1stExon", “TSS1500" or “TSS200" in the package ChAMPdata (version 2.28.0)]. To determine which probes are differentially methylated, *M* values were compared for each probe using Limma (version 3.52.0). *P* values were adjusted for multiple testing using the Benjamini–Hochberg (BH) procedure. Probes were considered hypermethylated in cancer when featuring an adjusted *P* value below 0.05, a logFC (*M*-value) in DNA methylation between normal and tumor samples of less than − 1, and an average difference in methylation (beta-value) of more than 10% to focus on biologically relevant results. Subsequently, differentially hypermethylated genes (HMGs) were defined as genes that have one or more hypermethylated probes within their promoter region. Hypomethylated probes and genes were defined analogously, i.e., with opposite effect size cut-offs. Genes featuring both hypermethylated and hypomethylated probes were included with both sets.

### Gene Ontology enrichment analysis and overrepresentation of neural terms within HMGs

GO analysis was performed on HMGs for each cancer type using the clusterProfiler (version 4.4.1) and ReactomPA packages (version 1.40.0, based on hypergeometric test). Enrichment was considered significant when the BH-adjusted P value was below 0.05. HMGs were compared against the background of all genes available on the Infinium Human Methylation 450k BeadChip array, and had probes located within their promoter region, similar to the criteria used in the differential analysis. HMGs were analyzed for enriched BPs, CCs, MFs, and Reactome pathways (PA).

GO terms related to the nervous system were identified using the following strings: “neuro”, “neuron”, “neuronal”, “neural”, “nervous”, “axon”, “dendritic”, “synaptic”, “synapse”, “learning”, “memory”, “brain”, “hippocampus” as previously performed [14]. Genes that were linked to at least one of these neural-related GOs were considered to be neural-related genes and referred to as such throughout this manuscript. GO annotation was retrieved from the Ensembl repository using the package biomaRt (version 2.52. 0). Frequencies of neural-related genes that are hypermethylated in their promoter region in each cancer type were calculated and compared to their frequency in the Infinium array. A Chi-squared test was applied to assess the enrichment of neural-related genes per cancer type. *P* values were corrected for multiple testing for the number of cancer types using the BH false discovery rate.

### Tumor purity

To rule out tumor purity as a confounder of the effect seen for neural-related HMGs, the correlation between tumor purity and average methylation levels for each sample was assessed. A list of consensus measurement of purity estimations (CPE) was retrieved for all TCGA samples [40]. The average methylation level was calculated for each sample for neural-related HMGs, non-neural-related HMGs, neural-related genes that were not differentially methylated and non-neural-related genes that were not differentially methylated. Robust linear regression was subsequently used to model the average methylation level per group as a function of tumor purity using the R package MASS (version 7.3.56).

### Bivalent genes

Bivalent genes were assessed as a potential confounder for the enrichment of neural-related genes within HMGs. Therefore, a list of bivalent genes in embryonic stem cells was retrieved from a previous study [41]. Pearson’s Chi-squared test was used to test the association between gene hypermethylation in cancer types according to their neural-related status against “bivalency”.

### Identifying neural differentiation genes

To identify genes associated with neural stemness and differentiation, we analyzed Affymetrix Human Gene 1.0 ST Array data [19] (available from the Gene Expression Omnibus, GSE65106). Differential expression was assessed between pluripotent stem cells (PSCs)-derived neurons compared to PSCs-derived neural progenitor cells using the Limma package (version 3.52.0). Genes that are overexpressed (adjusted for age and sex, with logFC > 1 and BH-adjusted *P* value < 0.05) in PSCs-derived neurons compared to PSCs-derived neural progenitor cells were classified as “differentiation-associated” genes.

### Differential expressed genes

To assess the impact of differential methylation on expression, differential expression analysis was performed. TCGA expression data were downloaded from GDC Xena hub as log scaled counts. The limma voom procedure was used to test for differential expression for each gene. Genes that have an absolute logFC in expression of more than 1 and a BH-adjusted *P* value < 0.05 were considered differentially expressed (DEGs).

### Cancer hallmarks analysis

For each gene set related to the hallmarks of cancer [15], enrichment for neural-related genes was tested, both in the HMGs and non-HMGs. Chi-squared tests were applied on the formulated contingency tables followed by BH-based *P* value adjustment.

### Upstream regulators analysis

The Ingenuity Pathway Analysis (IPA, Qiagen) software was used to map the neural-related COAD HMGs to upstream regulators. IPA upstream regulator analysis was performed on DNA methylation rather than the default gene expression data. As promoter DNA methylation is typically an expression silencing mark, we provided IPA with hypomethylated loci (probes within/near the promoter region, adjusted *P* value < 0.05, logFC (M-value) > 1, and absolute Δbeta-value > 10%) in the place of upregulated genes and hypermethylated (probes within/near the promoter region, adjusted *P* value < 0.05, logFC (*M*-value) < − 1, and absolute Δbeta-value > 10%) rather than downregulated loci. Next to evaluating enrichment of putatively regulated loci, IPA also tests for directionality (general activation or inhibition of those loci), of which the interpretation remains valid for the assumed DNA methylation–gene expression association. As a background, we considered all neural-related probes present on the Infinium array. Of the significant results, we filtered the output to solely retrieve terms labeled as “transcription regulator” with a BH-adjusted *P* value < 0.05.

### Supplementary Information

**Additional file 1**: The list of significantly differentially methylated probes is provided in the file “significant Limma output per cancer type.xlsx”. A list of all enriched GO terms per cancer type is provided in the file “GO outcome.rar”. A list of neural differentiation-associated genes is provided in the file “Neural differentiation-associated genes.xlsx”.

**Additional file 2**: **Table S1.** Enrichment for neural genes in hyper- and hypomethylated genes in CpG islands in solid cancers. **Table S2.** Pathways and GO term enrichment of hypermethylated genes over all solid cancer types. **Table S3**. Cross tables for hypermethylated (HMG) and non-hypermethylated (non-HMG) genes in COAD, considering bivalency and/or neural (GO) annotation. **Table S4.** Odds ratios of genes getting hypermethylated during cancer. **Table S5.** Ingenuity Pathway Analysis output on hypermethylated neural-related genes in COAD. **Figure S1.** Enrichment of neural terms in hypermethylated genes in cancer. **Figure S2.** Percentage of neural-related hyper- and hypomethylated genes in their promoter CpG islands in different solid cancers. **Figure S3.** Dotplot of the average methylation per sample in function of sample purity in COAD. **Figure S4.** Methylation levels of neural-related genes reflects overall methylation levels in COAD. **Figure S5.** Venn diagram for COAD HMGs, neural-related genes, and ESCs-derived bivalent genes.

## Data Availability

TCGA datasets analyzed during the current study can be freely and openly accessed from www.xenabrowser.net. GSE65106, GSE48684 and GSE156299 are publicly available on GEO. TCGA sample purity estimation [40], a list of bivalent genes in embryonic stem cells [41] was retrieved from the supplementary material of each article. Lists of genes involved in specific cancer hallmarks can be accessed through https://figshare.com/articles/dataset/Hallmarks_of_Cancer_Gene_Set_Annotation/4794025.

## Abbreviations

TCGA: The Cancer Genome Atlas
REST: RE1-Silencing Transcription factor
COAD: Colon adenocarcinoma
GO: Gene Ontology
TME: Tumor microenvironment
ESCs: Embryonic stem cells
iPSCs: Inducible pluripotent stem cells
PRC2: Polycomb repressive complex
BLCA: Bladder cancer
BRCA: Breast cancer
CHOL: Cholangiocarcinoma
UCEC: Endometrioid cancer
ESCA: Esophageal cancer
HNSC: Head and neck cancer
KIRC: Kidney clear cell carcinoma
KIRP: Kidney papillary cell carcinoma
LIHC: Liver cancer
LUAD: Lung adenocarcinoma
LUSC: Lung squamous cell carcinoma
PAAD: Pancreatic adenocarcinoma
PRAD: Prostate adenocarcinoma
READ: Rectal adenocarcinoma
THCA: Thyroid cancer
BH: Benjamini–Hochberg
FDR: False discovery rate
PSCs: Pluripotent stem cells
CPE: Consensus measurement of purity estimations
DEGs: Differentially expressed
IPA: Ingenuity Pathway Analysis
GEO: The Gene Expression Omnibus
