# Supplementary material for: Promoter hypermethylation of neural-related genes is compatible with stemness in solid cancers
Source: Epigenetics Chromatin. 2023 Aug 3;16:31. doi: 10.1186/s13072-023-00505-7 (PMC10398991; doi:10.1186/s13072-023-00505-7)

**Table S1: Enrichment for neural genes in hyper- and hypomethylated genes in CpG islands in solid cancers.** The number of differentially hyper- and hypomethylated genes and percentage of neural-related genes in CpG islands are shown for all solid cancers. The false discovery rate (FDR) column gives Benjamini-Hochberg adjusted P values for the enrichment of neural genes within hyper- and hypomethylation upon assessment with a chi-squared test, compared to the expected amount based on the Infinium array annotation.

| Cancer type | Number of hyper- methylated genes | Percentage of neural gene | FDR | Number of hypo-methylated genes | Percentage of neural gene | FDR |
| --- | --- | --- | --- | --- | --- | --- |
| BLCA | 1226 | 41.3 | 2.8e-50 | 490 | 20.2 | 0.74 |
| BRCA | 1800 | 39.0 | 1.1e-53 | 105 | 20.0 | 0.90 |
| CHOL | 1053 | 40.9 | 2.2e-42 | 229 | 29.7 | 0.08 |
| COAD | 2033 | 39.8 | 2.5e-64 | 82 | 18.3 | 0.83 |
| ESCA | 1055 | 39.4 | 2.7e-36 | 79 | 16.5 | 0.74 |
| HNSC | 1571 | 39.8 | 8.4e-53 | 125 | 16.8 | 0.74 |
| KIRC | 966 | 39.9 | 2.9e-35 | 86 | 24.4 | 0.91 |
| KIRP | 559 | 33.6 | 4.8e-10 | 119 | 21.0 | 0.91 |
| LIHC | 1436 | 38.7 | 4.8e-43 | 265 | 15.5 | 0.08 |
| LUAD | 1474 | 42.0 | 3.8e-62 | 90 | 23.3 | 0.91 |
| LUSC | 1183 | 35.9 | 3.2e-26 | 228 | 19.3 | 0.74 |
| PAAD | 1323 | 44.3 | 1.4e-69 | 103 | 23.3 | 0.91 |
| PRAD | 1664 | 35.2 | 1.9e-31 | 82 | 18.3 | 0.83 |
| READ | 1277 | 41.3 | 6.1e-52 | 251 | 18.7 | 0.74 |
| THCA | 82 | 36.6 | 0.003 | 37 | 18.9 | 0.91 |
| UCEC | 1353 | 37.3 | 4.2e-35 | 327 | 23.9 | 0.86 |

**Table S2: Pathways and GO term enrichment of hypermethylated genes over all solid cancer types. List of pathways and GO terms for which there was a significant enrichment in hypermethylated genes over at least 15 out of 16 cancer types. BP: biological process; CC: cellular component; MF: molecular function; PW: pathway.**

| Frequently enriched GO term | N cancers | ID | type |
| --- | --- | --- | --- |
| Neuronal System | 16 | R-HSA-112316 | PW |
| cartilage development | 15 | GO:0051216 | BP |
| anterior/posterior pattern specification | 15 | GO:0009952 | BP |
| cell-cell adhesion via plasma-membrane adhesion molecules | 15 | GO:0098742 | BP |
| cell fate commitment | 15 | GO:0045165 | BP |
| cell junction assembly | 15 | GO:0034329 | BP |
| connective tissue development | 15 | GO:0061448 | BP |
| dopaminergic neuron differentiation | 15 | GO:0071542 | BP |
| ear morphogenesis | 15 | GO:0042471 | BP |
| embryonic organ development | 15 | GO:0048568 | BP |
| embryonic organ morphogenesis | 15 | GO:0048562 | BP |
| embryonic skeletal system development | 15 | GO:0048706 | BP |
| embryonic skeletal system morphogenesis | 15 | GO:0048704 | BP |
| forebrain development | 15 | GO:0030900 | BP |
| inner ear morphogenesis | 15 | GO:0042472 | BP |
| mesenchymal cell differentiation | 15 | GO:0048762 | BP |
| mesenchyme development | 15 | GO:0060485 | BP |
| multicellular organismal signaling | 15 | GO:0035637 | BP |
| muscle contraction | 15 | GO:0006936 | BP |
| muscle organ development | 15 | GO:0007517 | BP |
| muscle organ morphogenesis | 15 | GO:0048644 | BP |
| muscle tissue development | 15 | GO:0060537 | BP |
| neuromuscular process | 15 | GO:0050905 | BP |
| pattern specification process | 15 | GO:0007389 | BP |
| positive regulation of neuron differentiation | 15 | GO:0045666 | BP |
| potassium ion transmembrane transport | 15 | GO:0071805 | BP |
| potassium ion transport | 15 | GO:0006813 | BP |
| regionalization | 15 | GO:0003002 | BP |
| regulation of blood pressure | 15 | GO:0008217 | BP |
| regulation of cation transmembrane transport | 15 | GO:1904062 | BP |
| regulation of cytosolic calcium ion concentration | 15 | GO:0051480 | BP |
| regulation of ion transmembrane transport | 15 | GO:0034765 | BP |
| regulation of membrane potential | 15 | GO:0042391 | BP |
| regulation of nervous system development | 15 | GO:0051960 | BP |
| regulation of neuron differentiation | 15 | GO:0045664 | BP |
| sensory organ morphogenesis | 15 | GO:0090596 | BP |
| skeletal system morphogenesis | 15 | GO:0048705 | BP |
| striated muscle tissue development | 15 | GO:0014706 | BP |
| axon terminus | 15 | GO:0043679 | CC |
| cation channel complex | 15 | GO:0034703 | CC |
| collagen-containing extracellular matrix | 15 | GO:0062023 | CC |
| distal axon | 15 | GO:0150034 | CC |
| integral component of postsynaptic membrane | 15 | GO:0099055 | CC |
| integral component of postsynaptic specialization membrane | 15 | GO:0099060 | CC |
| integral component of synaptic membrane | 15 | GO:0099699 | CC |
| intrinsic component of postsynaptic membrane | 15 | GO:0098936 | CC |
| intrinsic component of postsynaptic specialization membrane | 15 | GO:0098948 | CC |
| intrinsic component of synaptic membrane | 15 | GO:0099240 | CC |
| ion channel complex | 15 | GO:0034702 | CC |
| neuronal cell body | 15 | GO:0043025 | CC |
| perikaryon | 15 | GO:0043204 | CC |
| postsynaptic membrane | 15 | GO:0045211 | CC |
| postsynaptic specialization | 15 | GO:0099572 | CC |
| postsynaptic specialization membrane | 15 | GO:0099634 | CC |
| potassium channel complex | 15 | GO:0034705 | CC |
| synaptic membrane | 15 | GO:0097060 | CC |
| transmembrane transporter complex | 15 | GO:1902495 | CC |
| transporter complex | 15 | GO:1990351 | CC |
| voltage-gated potassium channel complex | 15 | GO:0008076 | CC |
| cation channel activity | 15 | GO:0005261 | MF |
| channel activity | 15 | GO:0015267 | MF |
| delayed rectifier potassium channel activity | 15 | GO:0005251 | MF |
| gated channel activity | 15 | GO:0022836 | MF |
| ion channel activity | 15 | GO:0005216 | MF |
| ligand-gated cation channel activity | 15 | GO:0099094 | MF |
| ligand-gated channel activity | 15 | GO:0022834 | MF |
| ligand-gated ion channel activity | 15 | GO:0015276 | MF |
| metal ion transmembrane transporter activity | 15 | GO:0046873 | MF |
| passive transmembrane transporter activity | 15 | GO:0022803 | MF |
| postsynaptic neurotransmitter receptor activity | 15 | GO:0098960 | MF |
| potassium channel activity | 15 | GO:0005267 | MF |
| potassium ion transmembrane transporter activity | 15 | GO:0015079 | MF |
| voltage-gated cation channel activity | 15 | GO:0022843 | MF |
| voltage-gated potassium channel activity | 15 | GO:0005249 | MF |
| Class A/1 (Rhodopsin-like receptors) | 15 | R-HSA-373076 | PW |
| G alpha (q) signaling events | 15 | R-HSA-416476 | PW |
| GPCR ligand binding | 15 | R-HSA-500792 | PW |
| Potassium Channels | 15 | R-HSA-1296071 | PW |
| Transmission across Chemical Synapses | 15 | R-HSA-112315 | PW |

**Table S3: Cross tables for hypermethylated (HMG) and non-hypermethylated (non-HMG) genes in COAD, considering bivalency and/or neural (GO) annotation.**

| Genes in COAD | HMG | Non-HMG | Total |
| --- | --- | --- | --- |
| Bivalent | 1,419 (54.07%) | 2,535 (10.67%) | 3,954 |
| Non-bivalent | 1,205 (45.92%) | 21,218 (89.33%) | 22,423 |
| Total | 2,624 | 23,753 | 26,377 |
| Bivalent, neural | 663 (46.72%) | 654 (25.80%) | 1,317 |
| Bivalent, non-neural | 756 (53.28%) | 1,881 (74.20%) | 2,637 |
| Total | 1,419 | 2,535 | 3,954 |
| Non-bivalent, neural | 314 (26.06%) | 2,446 (11.53%) | 2,760 |
| Non-bivalent, non-neural | 891 (73.94%) | 18,772 (88.47%) | 19,663 |
| Total | 1,205 | 21,218 | 22,423 |

**Table S4: Odds ratios of genes getting hypermethylated during cancer.** Column 1: odds ratios of hypermethylated genes being bivalent in embryonic stem cells (ESCs); Column 2: odds ratios of hypermethylated bivalent genes being neural-related; Column 3: odds ratios of hypermethylated non bivalent genes being neural-related. P values from chi-squared tests were FDR adjusted and reported following the key: FDR < 0.05: *; FDR < 10^-10^: **; FDR < 10^-50^: ***.

| Cancer type | Odds ratio of HMG being bivalent | Odds ratio of bivalent HMGs being neural-related | Odds ratio of non-bivalent HMGs being neural-related |
| --- | --- | --- | --- |
| BLCA | 6.13*** | 2.21** | 2.23** |
| BRCA | 6.16*** | 2.09** | 2.29** |
| CHOL | 4.54*** | 1.96** | 1.59** |
| COAD | 9.94*** | 2.35** | 2.72*** |
| ESCA | 4.94*** | 1.85** | 2.18** |
| HNSC | 6.27*** | 2.12** | 2.39** |
| KIRC | 4.42*** | 1.8** | 2.1** |
| KIRP | 2.06*** | 1.26* | 1.73** |
| LIHC | 7.38*** | 1.91** | 2.35** |
| LUAD | 8.25*** | 2.3** | 2.61** |
| LUSC | 5.03*** | 1.8** | 2.03** |
| PAAD | 8.86*** | 2.56** | 2.69** |
| PRAD | 4.79*** | 1.7** | 2.31*** |
| READ | 7.68*** | 2.29** | 2.33** |
| THCA | 3.63** | 1.28 | 1.9* |
| UCEC | 3.96*** | 2** | 1.88** |

**Table S5**: Ingenuity Pathway Analysis output on hypermethylated neural-related genes in COAD.

| **Upstream Regulator** | **Predicted Activation State** | **Bias-corrected z-score** | **Activation z-score** | **Adjusted *P* value** | **Target Molecules in Dataset** |
| --- | --- | --- | --- | --- | --- |
| **REST** | Activated | 0,425 | 5,632 | 2,63E-06 | ASCL1, ASCL2, B3GAT1, BDNF, CACNA1H, CACNG2, CARTPT, CBLN1, CHRNB2, CNTNAP1, CRH, CXCL12, EOMES, EPHA10, FGF12, FGF14, FGF5, FUT9, GAP43, GJD2, GLRA1, GRIA2, GRIK3, GRIN1, GRIN2A, INA, JAM2, KCNQ2, KCNQ3, LHX5, LIN28A, NCAM2, NEFH, NELL1, NEUROD1, NPTXR, NTRK3, OPRM1, PAX3, PENK, POU4F1, PTPRN, RGS7, SCTR, SLC12A5, SLC18A3, SNAP25, SRRM4, STMN2, SYN1, SYT1, TAC1, TBXT, UCHL1 |
| **TLX3** | Inhibited | 0,000 | -3,832 | 1,49E-04 | ASCL1, CCK, GABRA1, GABRB2, GALR1, GRIA2, GRIA3, GRIA4, GRIN3A, NEUROD1, NEUROG1, RET, SLC17A6, SLC18A3, SST, TAC1 |
| **POU4F1** |  | -0,178 | -0,674 | 2,76E-04 | ADCYAP1, BNC1, CARTPT, CHRNA3, CHRNB4, CNTN2, DCC, DMRTA2, EPHA8, GAL, GBX2, HOXC8, INA, IRX1, IRX2, IRX3, IRX6, ISL2, KCNMA1, KCTD8, LHX1, NEUROD1, NEUROD4, NOVA1, NPY, NTRK3, OLIG1, POU4F1, POU4F2, POU4F3, PRPH, RET, RGMA, RUNX3, SLC6A17, SNAP25, SSTR4, SYT9, TAC1, TFAP2B |
| **PTF1A** | Inhibited | 0,344 | -2,767 | 1,53E-03 | GAD2, GBX1, GBX2, GRIK1, GRIK2, GRIK3, GRM3, GSX2, ISL1, LAMP5, LHX1, LHX5, LMX1B, NPY, PAX2, PRDM13, SEZ6, SLC32A1, SLC6A5, SSTR2, TFAP2B, TLX3, UNCX |
| **POU4F2** |  | -0,853 | 0,853 | 1,03E-02 | BARHL2, BHLHE22, CALB1, DLX1, IRX4, LHX1, NEUROD1, NEUROD4, NEUROG2, OTX2, PAX6, POU4F1, POU4F2, POU4F3, PTF1A, SIX3, SLC32A1, SLC6A1, TFAP2A |
| **MESP1** | Inhibited | 0,000 | -3,162 | 1,10E-02 | FOXC2, HAND2, MEF2C, MEIS2, NKX2-5, PRICKLE1, SNAI2, TWIST1, ZEB1, ZEB2 |
| **NEUROG2** | Inhibited | -0,442 | -2,433 | 1,32E-02 | ASCL1, DOCK8, EMX2, EN1, FOXG1, GLI3, INSM2, IRX3, NEUROD1, NKX2-2, NKX6-1, NOG, OTX2, PAX2, PAX6, SOX3, TFAP2A, WNT1, WNT3A, ZIC1 |
| **PAX6** |  | 0,282 | -0,934 | 1,34E-02 | ASCL1, CDH8, CDK6, CELF4, CUX2, DBX1, EN1, EOMES, FAT4, FGF19, FUT9, GLP1R, GSX2, ISL1, KLHL1, NAV1, NCAM1, NEUROG1, NKX2-2, NKX6-1, NTN1, OTX2, PALM, PAX2, PAX3, PCSK1N, PCSK2, PDX1, SIX3, SNCA, SOX1, SST, TBR1, TNC |
| **GBX2** |  | -0,081 | -1,698 | 2,21E-02 | EGR2, EN1, ERC2, FGF12, FGF8, FOXG1, GRIA1, HOXA2, KCNB2, LAMA2, MAFB, OTX2, PAX2, PLXNA4, ROBO1, RTN1, SLIT3, SYT2, WNT1 |
| **CTNNB1** | Inhibited | -0,306 | -5,629 | 2,48E-02 | ABCD2, ADGRV1, ADRA2C, ALDH1A2, APC, AR, ASCL1, ASCL2, BCL2L11, BDNF, CACNA1G, CALM1 (includes others), CBLN1, CD34, CDH11, CDH2, CELSR1, CLDN5, CNR1, CNTFR, COL2A1, COL4A1, COL4A5, CTNNA2, CTNND2, CTSZ, CXCL12, DES, DKK1, DNAJC6, DTX1, ECRG4, EGFR, EMX2, EOMES, EPHA5, EVX1, F2R, FGF20, FGF5, FGF8, FN1, FOXB1, FOXC2, FZD1, FZD2, FZD7, GAP43, GATA2, GDNF, GHR, GLI3, GRIK1, GSC, HAPLN1, HDGF, HHIP, HMGCS2, HYCC1, ID4, IGF2BP1, INHA, IRF4, ISL1, ITGA4, JAM2, KDR, LEF1, LHCGR, LHX1, LHX6, LMX1B, MME, MMP2, MSX1, NCAM1, NDRG2, NES, NEUROD1, NEUROG1, NKX2-1, NKX2-5, NODAL, NPTX1, NRCAM, NRG1, NTN1, ODAD2, OLIG1, ONECUT2, PAX3, PDE1C, PDE4B, PIK3R1, PITX2, PMP22, POU3F2, PTCH1, PTGS2, PTPRJ, SALL1, SDC2, SDHA, SFRP1, SGK1, SIM2, SIX1, SLC1A2, SLC6A1, SLC6A2, SMO, SNAI2, SOX11, SOX17, SOX5, SYNM, TBR1, TBX20, TBX21, TBXT, TCF4, TGFB3, TNC, TP73, TUBB2B, TWIST1, VCAN, WNT1, WNT10A, WNT16, WNT2, WNT3, WNT3A, WNT7A, WNT9A, ZEB1, ZEB2, ZIC1, ZIC5 |
| **MESP2** | Inhibited | 0,000 | -3,162 | 2,48E-02 | FOXC2, HAND2, MEF2C, NKX2-5, PAX3, SNAI2, TWIST1, UNCX, ZEB1, ZEB2 |
| **ISL1** |  | 0,146 | -1,569 | 2,48E-02 | ABCC8, ADCYAP1, BNC1, CARTPT, CNTN2, DCC, DMRTA2, GAL, GBX2, HOXC8, IRX3, ISL2, LHX1, MSX1, NEUROD1, NEUROD4, NOVA1, NPY, NTRK3, OLIG1, PAX6, PCSK2, PDX1, POU4F1, POU4F2, POU4F3, PRPH, RET, RUNX3 |
| **TSHZ3** |  | 0,872 | 0,706 | 3,10E-02 | ACVR1C, ADCY1, BHLHE22, FEZF2, GSG1L, IGSF21, KCTD12, KLHL1, LDB2, NPY, NTRK3, RSPO2, SLC1A2, SLITRK3, TRPC7 |

**Figure S1: Enrichment of neural terms in hypermethylated genes in cancer.** Panel A shows an enrichment map plot of GO enriched terms for hypermethylated genes in their promoter CpG islands in COAD. Panel B shows a tree plot of the top Reactome pathways enriched for hypermethylated genes in their promoter CpG islands in COAD.


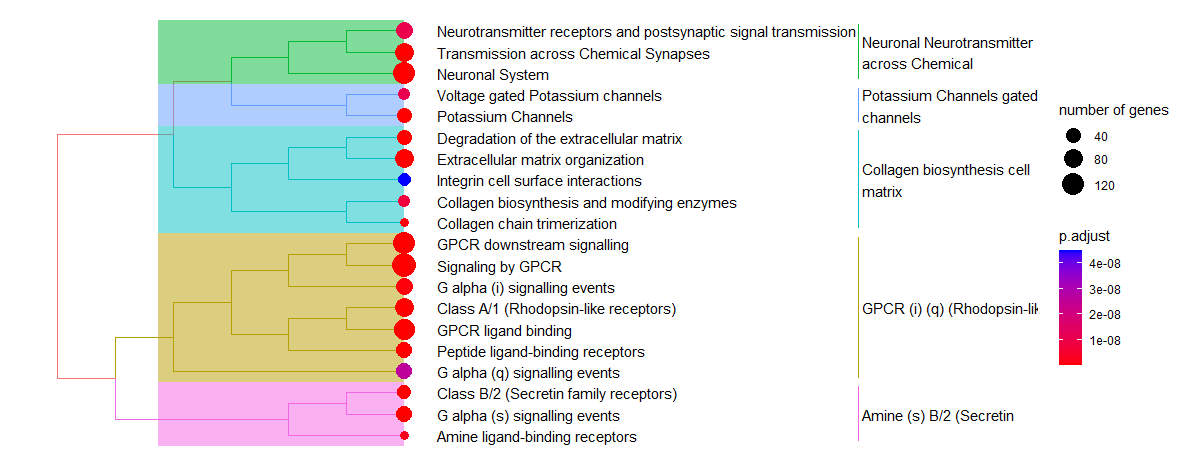
A B


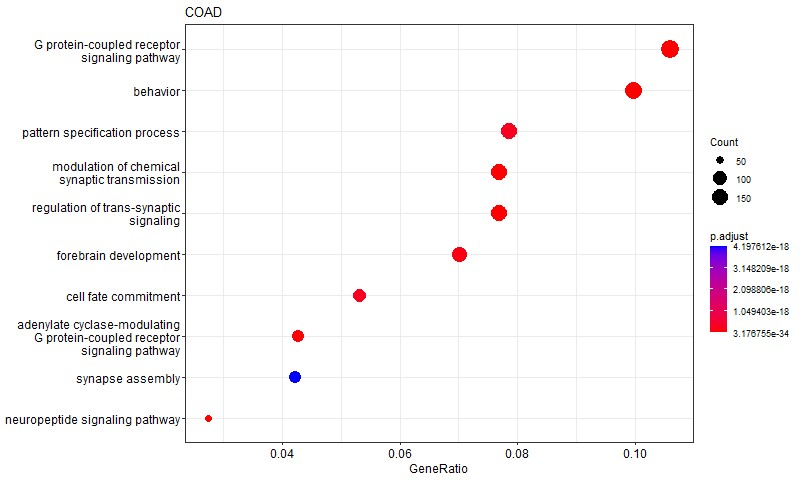


**Figure S2: Percentage of neural related hyper- and hypomethylated genes in their promoter CpG islands in different solid cancers.** Percentage of neural-related hypermethylated (left) and hypomethylated (right) genes in their promoter CpG islands for all analyzed solid cancer types. A paired two-tailed t-test was used to compare percentages of neural-related hypomethylated and hypermethylated genes.


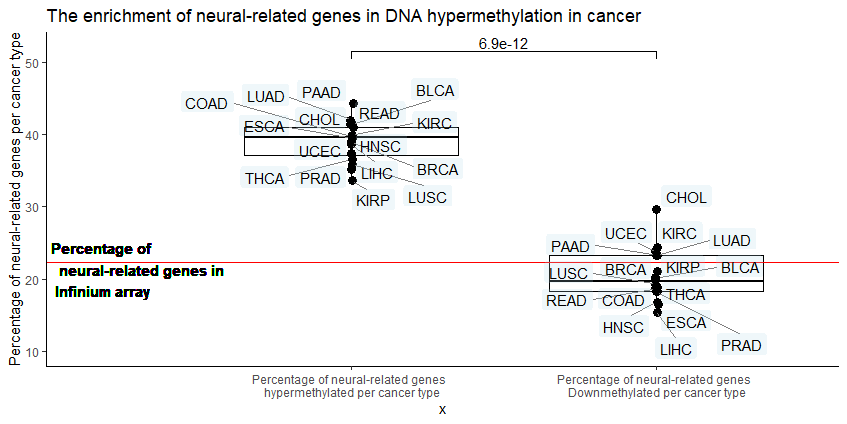


**Figure S3: Dotplot of the average methylation per sample in function of sample purity in COAD.**


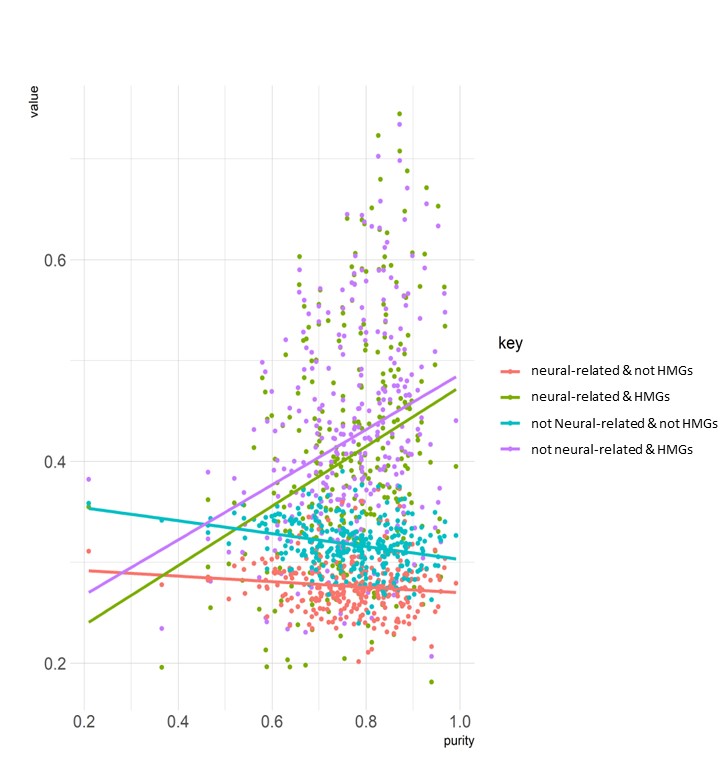


**Figure S4: Methylation levels of neural-related genes reflects overall methylation levels in COAD.** The red line represents the mean beta value of neural-related genes from each patient in COAD dataset, while the blue dots represent the mean beta values of non-neural genes for the same patients in the exact order as the red line.


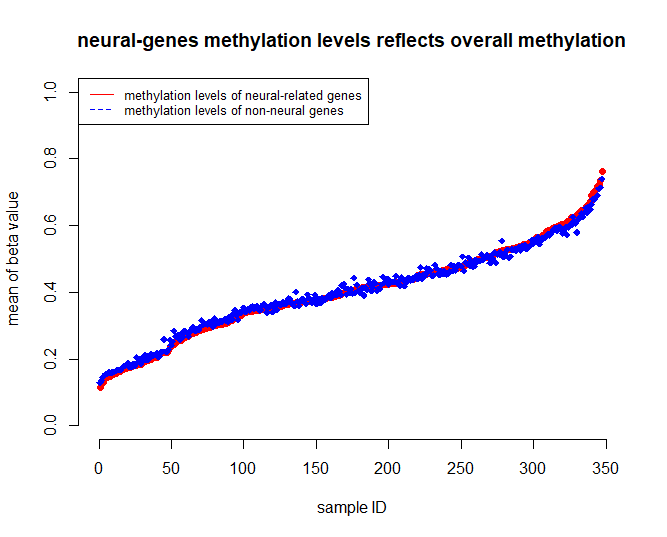


**Figure S5: Venn diagram for COAD HMGs, neural-related genes, and ESCs-derived bivalent genes.**


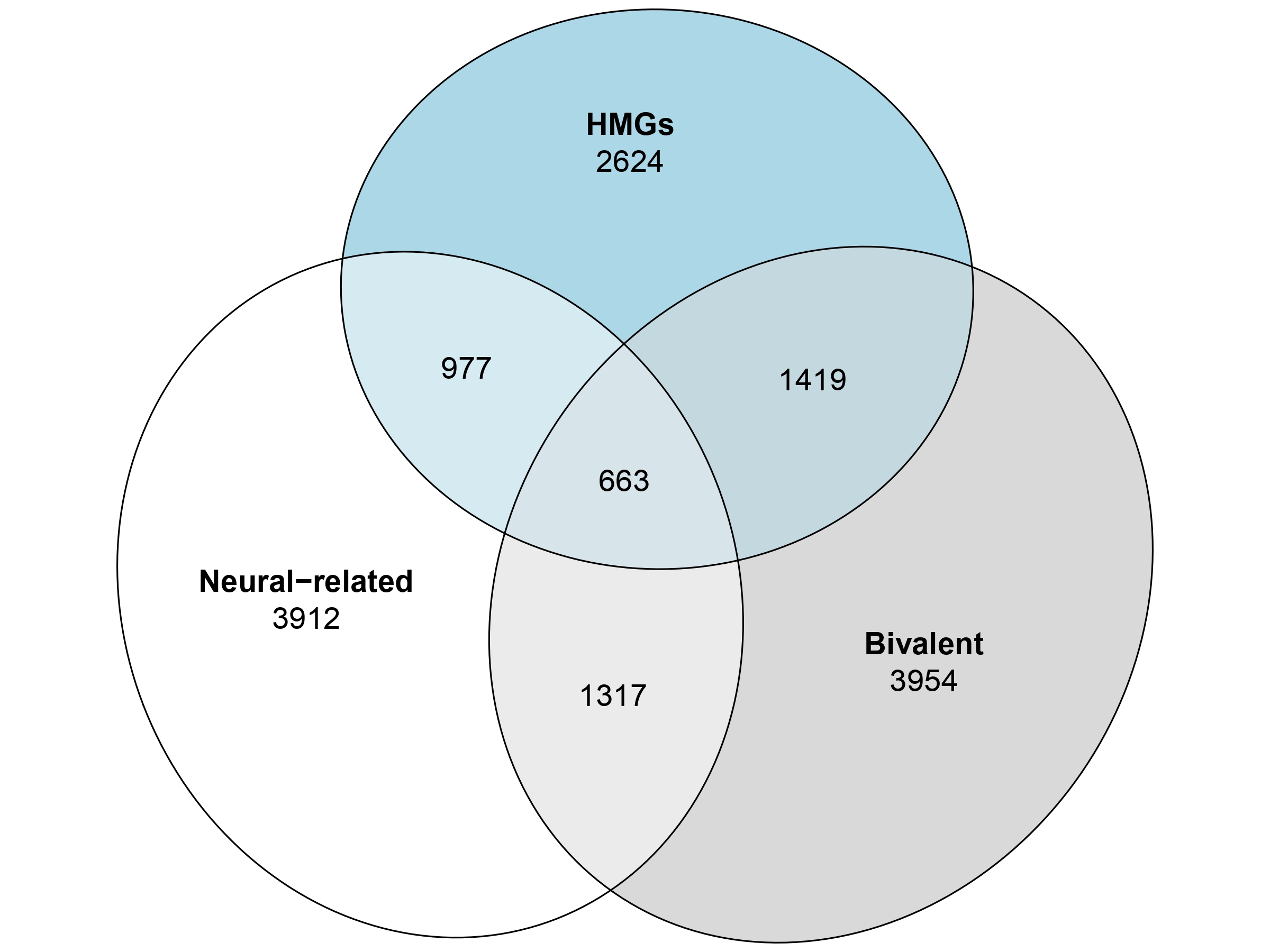

Supplement: Supplementary file 2 — Additional file 2: Table S1. Enrichment for neural genes in hyper- and hypomethylated genes in CpG islands in solid cancers. Table S2. Pathways and GO term enrichment of hypermethylated genes over all solid cancer types. Table S3. Cross tables for hypermethylated (HMG) and non-hypermethylated (non-HMG) genes in COAD, considering bivalency and/or neural (GO) annotation. Table S4. Odds ratios of genes getting hypermethylated during cancer. Table S5. Ingenuity Pathway Analysis output on hypermethylated neural-related genes in COAD. Figure S1. Enrichment of neural terms in hypermethylated genes in cancer. Figure S2. Percentage of neural-related hyper- and hypomethylated genes in their promoter CpG islands in different solid cancers. Figure S3. Dotplot of the average methylation per sample in function of sample purity in COAD. Figure S4. Methylation levels of neural-related genes reflects overall methylation levels in COAD. Figure S5. Venn diagram for COAD HMGs, neural-related genes, and ESCs-derived bivalent genes. [file 13072_2023_505_MOESM2_ESM.doc]
